# Overexpression of Synaptopodin increases the number of spine apparatuses and active synapses of dentate granule cells

**DOI:** 10.1038/s41598-026-57346-z

**Published:** 2026-06-11

**Authors:** Domenico Del Turco, Mandy H. Paul, Michael Rietsche, Jessica Schlaudraff, Alois Kreuzer, Martin Mittag, Alexander Drakew, Peter Jedlicka, Carlos Bas-Orth, Jochen Roeper, Thomas Deller

**Affiliations:** 1 Institute of Clinical Neuroanatomy, Dr. Senckenberg Anatomy, Neuroscience Center, Goethe-University Frankfurt, Frankfurt am Main, Germany; 2 Institute of Neurophysiology, Neuroscience Center, Goethe-University Frankfurt, Frankfurt am Main, Germany; 3 ICAR3R-Interdisciplinary Centre for 3Rs in Animal Research, Faculty of Medicine, Justus Liebig University Giessen, Giessen, Germany; 4 Frankfurt Institute for Advanced Studies, Frankfurt am Main, Germany; 5 Present Address: Department of Diagnostic and Interventional Radiology, Marienhospital, Stuttgart, Germany

**Keywords:** Hippocampus, Dentate gyrus, Dendritic spines, Endoplasmic reticulum, Neuronal ensembles, Structural plasticity, Cell biology, Neuroscience

## Abstract

Synaptopodin (SP) is a plasticity-related actin-modulating protein found in a subpopulation of mature cortical spines. Its presence within spines is essential for the formation of the spine apparatus (SA) organelle, a local calcium store. SP has been functionally linked to enhanced synaptic strength and long-term spine stability, i.e., indicators of potentiated synapses involved in memory trace formation. As SP is transcriptionally upregulated under contextual learning conditions in dentate granule cells (GCs), we generated a gain-of-function mouse model (Cyan Fluorescent Protein-SP transgenic mouse; CSPtg) with a ~threefold overexpression of SP to study the cellular effects of an increased availability of SP in GCs. Like the wild-type (WT) protein, transgenic SP was sorted to spines and rescued the SA organelle in GCs of SP-deficient mice. In CSPtg mice, SP was found in twice as many spines compared to WT. At the ultrastructural level, a ~threefold increase in the number of SAs was observed. Other structural properties, including spine density, average spine head size, and average SP puncta size, were not significantly altered. Patch-clamp recordings of GCs in CSPtg mice revealed an ~ 3.5-fold increase in miniature excitatory postsynaptic current frequency, indicating that the increased expression of SP/SA in spines resulted in a larger number of active synapses. Thus, higher levels of SP in GCs may increase the number of synapses available for strengthening and plasticity. This could enhance the ability of GCs to be recruited into new ensembles under contextual learning conditions.

**Supplementary Information:**

The online version contains supplementary material available at 10.1038/s41598-026-57346-z.

## Introduction

Dendritic spines are postsynaptic elements of glutamatergic synapses in the brain^1,2^. The size of the spine head positively correlates with the strength of the axospinous synapse and the density of the postsynaptic α-amino-3-hydroxy-5-methylisoxazole-4-propionic acid (AMPA) subtype of glutamate receptors (AMPARs)^3–6^. In addition to the surface geometry of a spine, the strength of excitatory synapses depends on organelles within the dendritic spine. In particular, the presence of endoplasmic reticulum (ER) plays an important role for synaptic adaptations and is important for the expression of different forms of synaptic plasticity^7–12^. Mechanistically, spine ER acts as a calcium store^9,13^, with its presence influencing the trafficking and synthesis of postsynaptic molecules^9,14–19^. Within spines, ER can be either loosely organized or densely stacked^9^. In the latter case, several tubules or cisterns of ER are connected by a complex dense matrix^20,21^, forming the so-called spine apparatus (SA) organelle^22,23^.

The first protein identified as an essential component of the SA was Synaptopodin (SP)^24,25^, an actin-modulating protein found in kidney podocytes and telencephalic neurons^26,27^. Approximately 10% of hippocampal spines, depending on the hippocampal subfield and layer, contain SP and a SA organelle^28,29^. Direct comparison of the synaptic strength of SP-positive (SP+) and SP-negative (SP-) spines of equal size using glutamate uncaging and patch-clamp recordings revealed that SP+ spines have larger miniature excitatory postsynaptic currents (mEPSCs)^15^ and exhibit larger calcium transients^30,31^. In addition, SP+ spines are more stable than SP- spines of equal size^29^, as SP stabilizes actin bundles and links them to the ER ^20^, and may interact with the stable pool of actin forming the central core of spines^32–36^. In situ hybridization revealed a somatic expression of *SP* mRNA^25,27^, indicating that SP is primarily synthesized in the cell body and subsequently transported to its target compartments. Although most neurons express SP constitutively, its transcription is upregulated under conditions of synaptic activation^37,38^. Of particular interest in this context is the behavioral upregulation of *SP* mRNA and SP protein in granule cell (GC) ensembles encoding specific environmental contexts^39^. These neuronal ensembles in the dentate gyrus (DG) also express activity-regulated cytoskeleton-associated protein (Arc)^40,41^, which is even more abundantly expressed under conditions of SP-deficiency^42^. Although these findings link SP upregulation in GCs to contextual learning, the structural and functional consequences of this increased availability of SP in single GCs remain unclear. To address this question in adult mice in vivo, we generated a Cyan Fluorescent Protein (CFP)-SP overexpressing mouse line (CSPtg). This gain-of-function approach revealed that higher levels of SP are sufficient to increase the number of SP+ spines that contain SA organelles. Patch-clamp recordings from GCs further revealed an increase in mEPSC frequency in CSPtg mice, suggesting that SP availability influences the pool of active synapses in hippocampal neurons. Thus, SP upregulation in behaviorally activated GCs may increase the number of synapses available for plasticity and rewiring and may contribute to their integration into novel ensembles.

## Results

### SP is overexpressed in the hippocampus of adult CSPtg mice

**Fig. 1:**
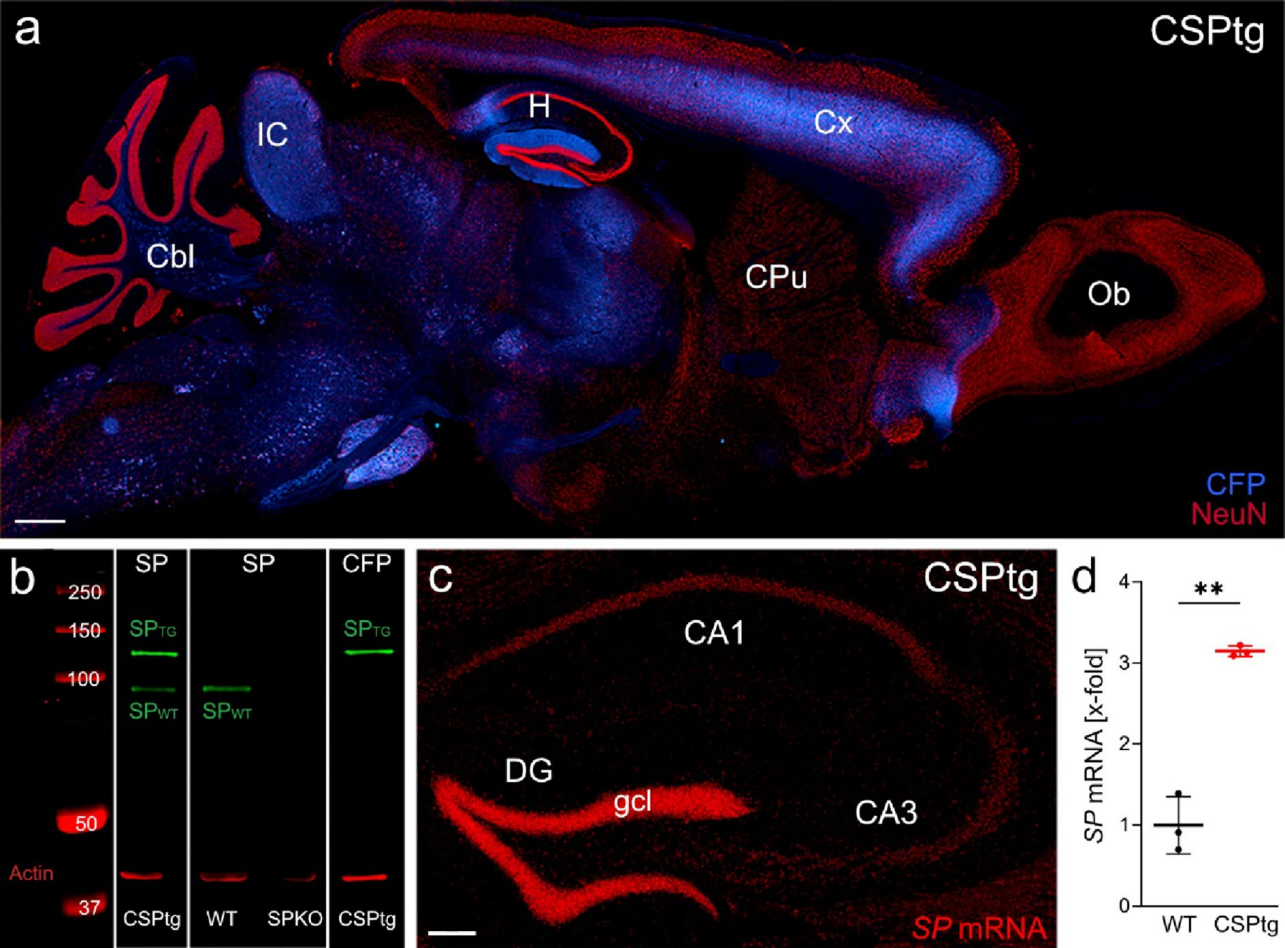
Characterization of the Cyan Fluorescent Protein-Synaptopodin transgenic mouse line. **(a)** Expression of Cyan Fluorescent Protein (CFP)-Synaptopodin (SP) in the brain of a CFP-SP transgenic mouse (CSPtg). Note the bright CFP fluorescence in the hippocampus (H). Neuronal nuclei marker (NeuN) was used for counterstaining. Cbl: cerebellum; Cx: cortex; CPu: striatum; IC: inferior colliculus; Ob: olfactory bulb. **(b)** Qualitative western blot analysis of CSPtg, wild-type (WT), and SP-deficient (SPKO) hippocampal tissue. Using an SP-specific antibody, wild-type SP (SP_WT_) was detected at ~ 95 kDa in WT and CSPtg mice, while transgenic SP (SP_TG_) was detected at ~ 120 kDa. SP protein was absent from the hippocampus of SPKO mice. Using an antibody for CFP, only SP_TG_ was detected in CSPtg. β-actin (Actin) was used as a loading control. Blots were cropped for presentation and arranged using white space to indicate non-adjacent lanes and/or separate blots. Full-length original blots are provided in the Supplementary Information. **(c)** Fluorescence in situ hybridization of *SP* mRNA in the hippocampus of a CSPtg mouse. Note the strong signal in the granule cell layer (gcl) of the dentate gyrus (DG) compared to the weaker signals in the cornu ammonis (CA) regions CA1 and CA3 of the hippocampus. **(d)** Scatter plot shows the relative difference in *SP* mRNA expression in the CSPtg compared to WT hippocampus, as measured by quantitative PCR (Unpaired t-test with Welch’s correction: *p* = 0.0073; *N* = 3 mice/genotype). Scale bars: (a) 500 μm, (c) 100 μm.

Adult CSPtg mice showed bright fluorescence in most telencephalic regions of the brain (Fig. 1a). This was particularly evident in cortical areas and in the hippocampus, especially in the DG. Prominent fluorescence was also observed in some diencephalic regions and brainstem nuclei, such as the inferior colliculus. Qualitative western blot analysis of hippocampal tissue from CSPtg mice using antibodies against SP and CFP verified the expression of wild-type SP (SP_WT,_ ~ 95 kDa) and transgenic CFP-SP (SP_TG,_ ~ 120 kDa) (Fig. 1b). As a specificity control, the antibody failed to detect SP_WT_ protein in the hippocampus of SP-deficient (SPKO) mice. Additionally, only SP_TG_ was detected in CSPtg mice using an antibody against CFP (Fig. 1b). Fluorescence in situ hybridization for *SP* mRNA revealed a strong signal in the dentate granule cell layer (gcl) of the CSPtg mouse (Fig. 1c). Quantitative PCR demonstrated that *SP* mRNA expression is approximately threefold higher in the hippocampus of CSPtg than in wild-type (WT) mice (Fig. 1d). Thus, SP_TG_ is robustly expressed in the hippocampus of adult CSPtg mice, with significantly higher SP expression levels than in WT controls.

### The distribution pattern of SP_TG_ in the hippocampus of adult CSPtg mice resembles that of SP_WT_

**Fig. 2:**
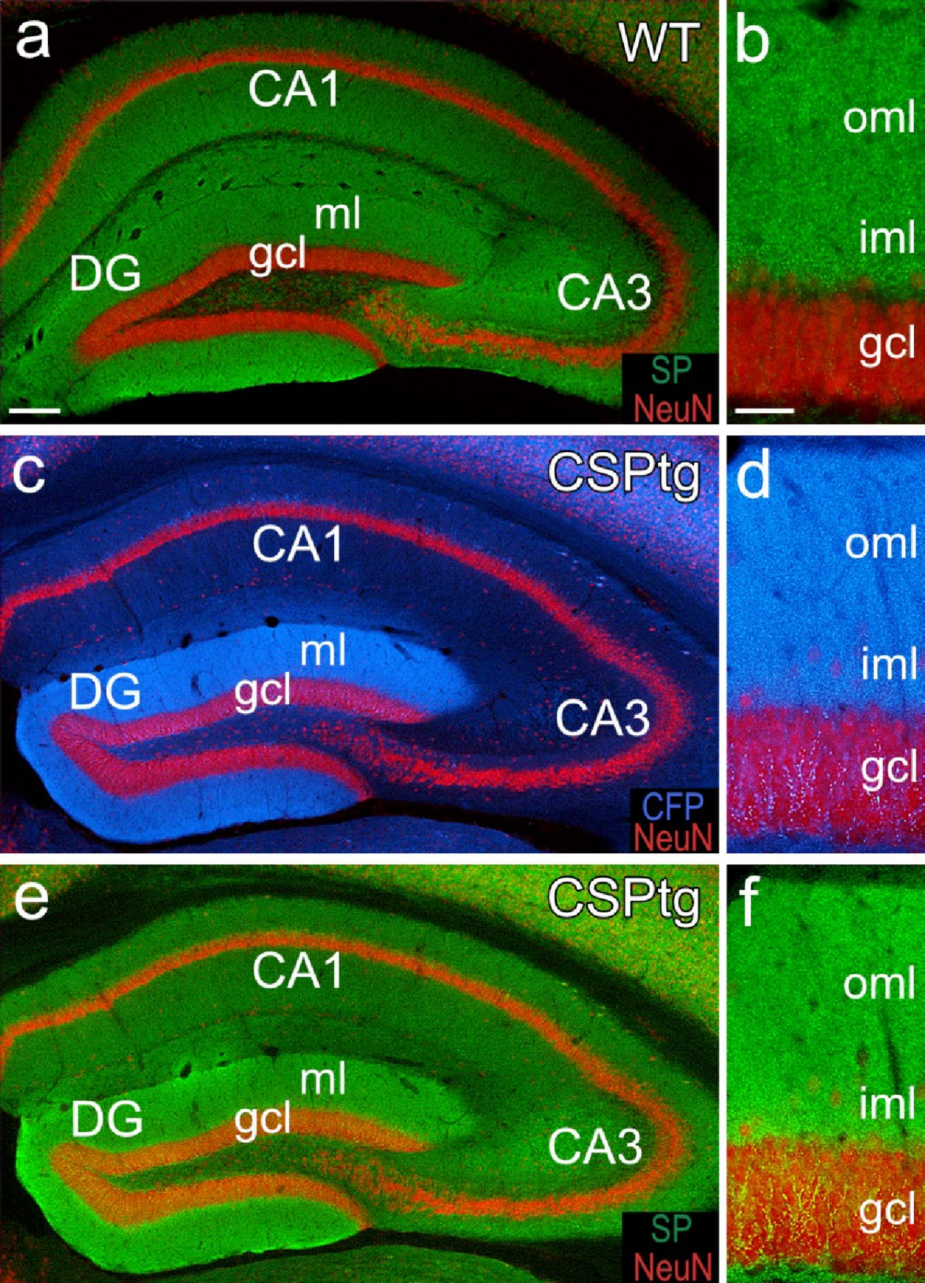
Synaptopodin is overexpressed in the dentate gyrus of Cyan Fluorescent Protein-Synaptopodin transgenic mice. **(a)** Immunofluorescence for Synaptopodin (SP) and NeuN in the hippocampus of a wild-type (WT) mouse. **(b)** Higher magnification of the dentate gyrus (DG). SP_WT_ puncta are abundant in the outer molecular layer (oml) and the inner molecular layer (iml). These puncta correspond to spine apparatus organelles in dendritic spines of dentate granule cells (GCs). Fewer SP puncta are found in the granule cell layer (gcl). These puncta correspond to cisternal organelles located in the axon initial segment of GCs. **(c)** Native Cyan Fluorescent Protein (CFP) fluorescence (SP_TG_) in the hippocampus of a CFP-SP transgenic mouse (CSPtg). Section is counterstained with the neuronal marker NeuN. Note the bright fluorescence in the molecular layer (ml) of the DG compared to the weaker fluorescence in cornu ammonis (CA) areas CA1 and CA3. **(d)** Higher magnification of the DG reveals the characteristic punctate pattern of SP in the oml, iml, and gcl. **(e)** Immunofluorescence for SP (visualizes both SP_WT_ and SP_TG_) and NeuN in hippocampus of the CSPtg mouse. **(f)** Higher magnification of the DG demonstrating the characteristic pattern of SP in all three layers. Scale bars: (a, c, e) 100 μm; (b, d, f) 25 μm.

Next, we examined the distribution pattern of SP_TG_ in the hippocampus of CSPtg mice and compared it to the well-described distribution pattern for SP_WT_ in WT mice (Fig. 2). In WT animals, SP puncta in the dentate molecular layer (ml) are associated with the SA organelle^25,47^, while SP puncta in the gcl primarily associate with the cisternal organelle of the axon initial segment^48^ (Fig. 2a, b). SP_TG_ fluorescence revealed a similar pattern in the hippocampus of CSPtg mice (Fig. 2c, d). However, compared to WT mice, where SP_WT_ immunofluorescence is comparable in the DG and Cornu Ammonis (CA) (Fig. 2a), SP_TG_ fluorescence was much stronger in the DG (Fig. 2c). Using an antibody against SP, we labeled both SP_WT_ and SP_TG_ in the hippocampus of the CSPtg mouse, visualizing the distribution of total SP (Fig. 2e, f). Based on these observations, we conclude that the distribution pattern of SP_TG_ puncta in the CSPtg mouse is similar to that of SP_WT_, and that the transgene is differentially expressed within the hippocampus, with higher expression levels in the DG than in CA.

### SP is localized in dendritic spines of GCs in CSPtg mice

**Fig. 3:**
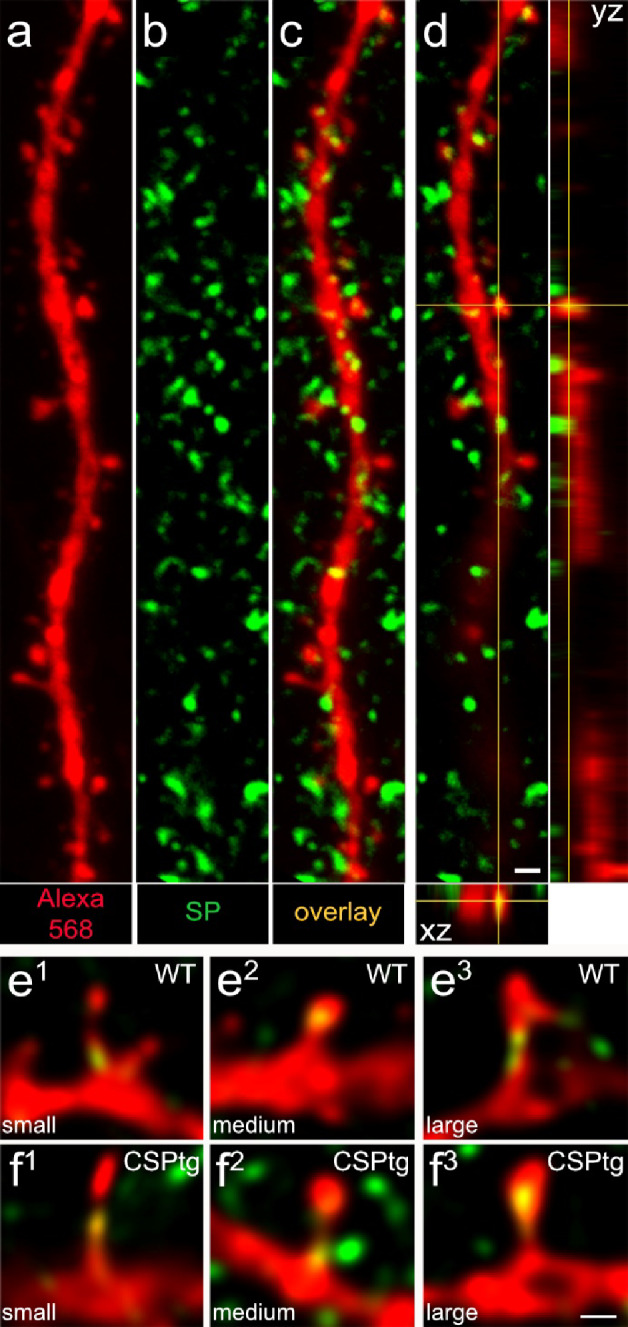
Synaptopodin is localized in dendritic spines of granule cells in the Cyan Fluorescent Protein-Synaptopodin transgenic mouse. **(a)** Confocal maximum projection of an Alexa568-filled dendritic segment of a granule cell (GC) in the dentate molecular layer of a Cyan Fluorescent Protein-Synaptopodin transgenic mouse (CSPtg). **(b)** Synaptopodin (SP)-antibody staining of the same dendritic segment. **(c)** Overlay image. **(d)** Cross-sectional views of a single optical section taken from the same dendritic segment. A large spine head containing SP is marked (cross-line, yellow). **(e1-3)** Images of small (e1), medium (e2) and large (e3) SP-positive (SP+) GC spines in the wild-type (WT) mouse. **(f1-3)** Images of small (f1), medium (f2) and large (f3) SP+ GC spines in the CSPtg mouse. Scale bars: (a-d) 1 μm; (e1-f3) 0.5 μm.

Next, we investigated the cellular localization of SP puncta in the DG ml of CSPtg mice. For this, we filled single GCs with Alexa-568 to visualize their dendrites and spines, and we combined it with SP antibody labeling. Similar to WT mice^29^, single SP puncta were found in dendritic spines in the DG ml (Fig. 3). Furthermore, SP was present in spines with different head sizes in both WT (Fig. 3e1-e3) and transgenic GCs (Fig. 3f1-f3).

### SP overexpression increases the fraction of SP-positive spines in GCs

**Fig. 4:**
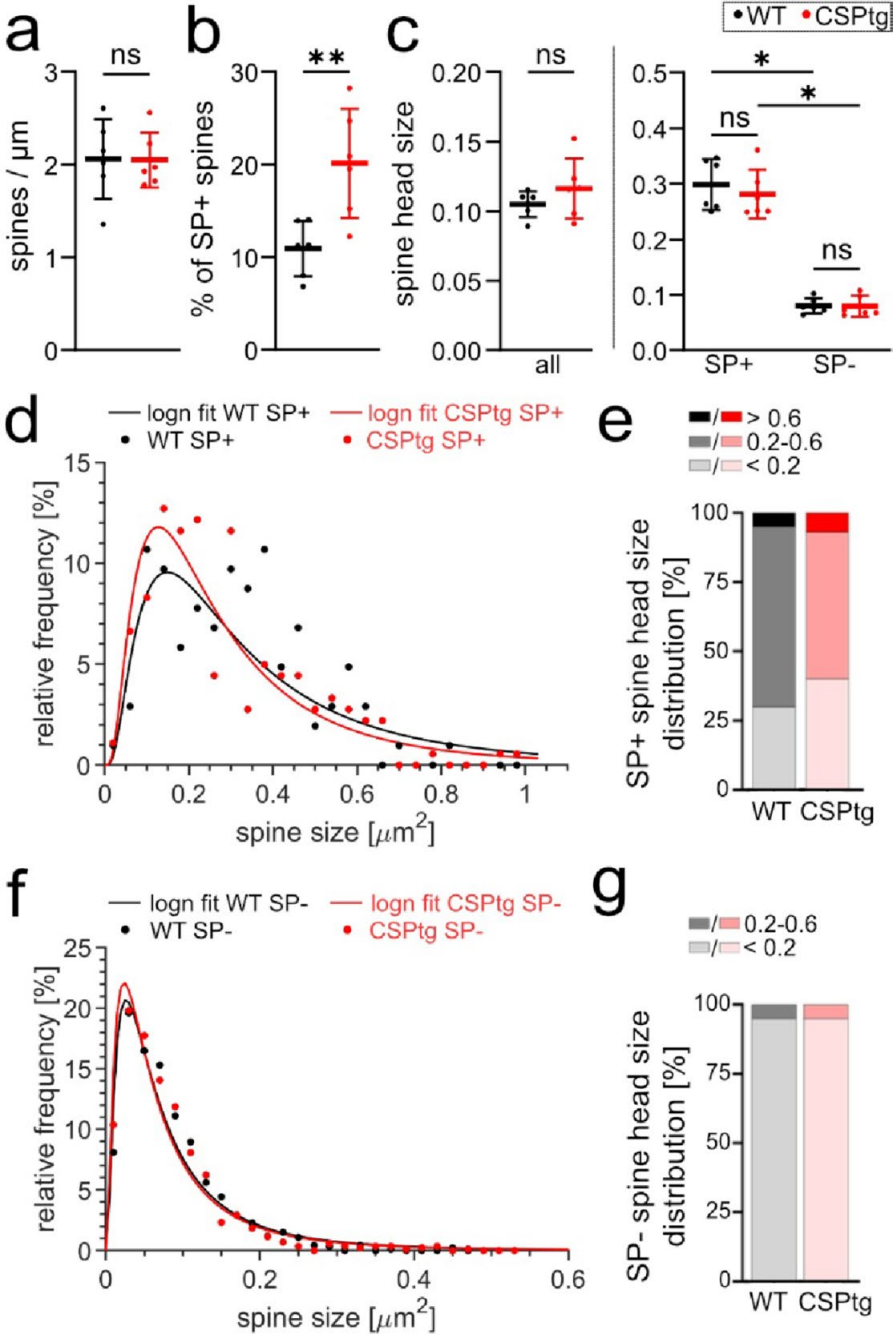
Granule cells in Cyan Fluorescent Protein-Synaptopodin transgenic mice have an increased number of Synaptopodin-positive spines. **(a)** Spine density was similar in wild-type (WT) and Cyan Fluorescent Protein-Synaptopodin (SP) transgenic (CSPtg) animals (Mann-Whitney (M-W) test: not significant (ns); *N* = 6 mice/genotype). **(b)** The percentage of SP-positive (SP+) spines was significantly increased in CSPtg compared to WT mice (M-W test: *p* = 0.0087; *N* = 6 mice/genotype). **(c)** Spine head size analysis was comparable for all spine types (M-W test: ns; *N* = 6 mice/genotype) and for the fractions of SP-positive (SP+) and SP-negative (SP-) spines (Kruskal-Wallis (K-W) with Dunn’s multiple comparisons test: ns; *N* = 6 mice/genotype). In contrast, significant differences were seen between spine head sizes of SP+ and SP- spines in both WT and CSPtg mice (K-W with Dunn’s multiple comparisons test: *p* = 0.0132; *N* = 6 mice/ genotype). **(d)** Histograms of SP+ spines in WT and CSPtg mice. Both datasets follow lognormal-like distributions with Lognormal(µ, σ^2^)_WT SP+_ = Lognormal(-1.2504, 0.8111) and Lognormal(µ, σ^2^)_CSPtg SP+_ = Lognormal(-1.4491, 0.7838). Goodness-of-fit values are r^2^_WT SP+_ = 0.64 and r^2^_CSPtg SP+_ = 0.9. Distributions show a trend towards a difference between genotypes (Kolmogorov-Smirnov (K-S) test: *p* = 0.0504). **(e)** Descriptive analysis of SP+ spine head sizes in WT and CSPtg mice (*N* = 6 mice/genotype; WT: *n* = 103 spines; CSPtg: *n* = 182 spines). Compared to WT, CSPtg mice show more small (< 0.2 μm²), fewer medium (0.2–0.6 μm²) and some more large (> 0.6 μm²) SP+ spines. **(f)** Histograms of SP- spines in WT and CSPtg mice. Both datasets follow very similar lognormal-like distributions with Lognormal(µ, σ^2^)_WT SP−_ = Lognormal(-2.7515, 0.9402) and Lognormal(µ, σ^2^)_CSPtg SP−_ = Lognormal(-2.8203, 0.9571); K-S test: ns. Goodness-of-fit values are r^2^_WT SP−_ = 0.94 and r^2^_CSPtg SP−_ = 0.93. **(g)** Descriptive analysis of SP- spine head sizes in WT and CSPtg mice (*N* = 6 mice/genotype; WT: *n* = 928 spines; CSPtg: *n* = 868 spines). WT and CSPtg show essentially identical distributions for small and medium spines, while large spines are absent.

Using Alexa568-filled GCs immunolabeled for SP, we determined the spine densities, the fraction of SP-positive (SP+) and SP-negative (SP-) spines, and their average spine head sizes (Fig. 4a-c). With regard to spine densities (Fig. 4a), there was no significant difference between WT (2.06 ± 0.43 spines/µm) and CSPtg (2.05 ± 0.29 spines/µm). This finding is in line with our previous observation that young adult SPKO mice show normal spine densities^24^, indicating that alterations in SP expression levels do not impact this parameter. However, we detected a significant, approximately twofold increase in the proportion of SP+ spines (Fig. 4b) in CSPtg (20.14 ± 5.9%) compared to WT mice (10.94 ± 2.98%). The average spine head sizes (Fig. 4c) of all spines (WT: 0.105 ± 0.009 μm² vs. CSPtg: 0.116 ± 0.021 μm²), SP+ spines (WT: 0.30 ± 0.05 μm² vs. CSPtg: 0.28 ± 0.04 μm²), and SP- spines (WT: 0.081 ± 0.013 μm² vs. CSPtg: 0.080 ± 0.019 μm²) were comparable and not significantly different. Since SP is primarily found in large spines^15,29,49^, we examined the spine head size of SP+ and SP- spines in WT and CSPtg mice. Consistent with these previous studies, SP+ spines were significantly larger than SP- spines in both WT and CSPtg mice (Fig. 4c).

### SP+ and SP- spine head sizes follow lognormal distributions

We then examined the lognormal (logn) distributions of SP+ and SP- spine head sizes of GCs in WT and CSPtg. The SP+ spine head distributions (Fig. 4d) did not reach statistical significance (K-S test: *p* = 0.0504), but showed a trend towards a difference between the two genotypes (WT: logn (-1.2504, 0.8111), r^2^ = 0.64 vs. CSPtg: logn (-1.4491, 0.7838), r^2^ = 0.9). In contrast, the distributions of SP- spines (Fig. 4f) were very similar between genotypes (WT: logn (-2.7515, 0.9402), r^2^ = 0.94 vs. CSPtg: logn (-2.8203, 0.9571), r^2^ = 0.93). Additional comparisons within each genotype showed that SP+ and SP− spine head size distributions differed significantly in both WT mice (K-S test: *p* < 0.001) and CSPtg mice (K-S test: *p* < 0.001). Thus, SP− spine head sizes were concentrated more strongly in the small size range, whereas SP+ spine head sizes were distributed more widely into the medium and large size ranges.

### Spine head size distribution of SP+ spines

To describe the distribution of spine head sizes more closely, we subdivided SP+ and SP- spines into three categories: small (s; < 0.2 μm²), medium (m; 0.2–0.6 μm²), and large (l; > 0.6 μm²) spines. This descriptive analysis revealed that SP overexpression does not simply increase the fraction of SP+ spines uniformly. Rather, CSPtg mice showed a higher fraction of small SP+ spines (WT: 30% vs. CSPtg: 40%; Fig. 4e) and a lower fraction of medium-sized spines (WT: 65% vs. CSPtg: 53%; Fig. 4e). We also observed a small increase in the fraction of large SP+ spines (WT: 5% vs. CSPtg: 7%; Fig. 4e). In contrast, SP- spines categories were essentially identical between WT and CSPtg mice (WT, CSPtg: s (95%), m (5%), l (0%); Fig. 4g). Thus, the altered category distribution was restricted to the SP+ spine population and was mainly reflected by a shift toward smaller SP+ spine sizes, whereas SP− spines remained unaffected.

### The relationship between SP puncta size and spine head size is maintained in GCs of CSPtg mice

**Fig. 5:**
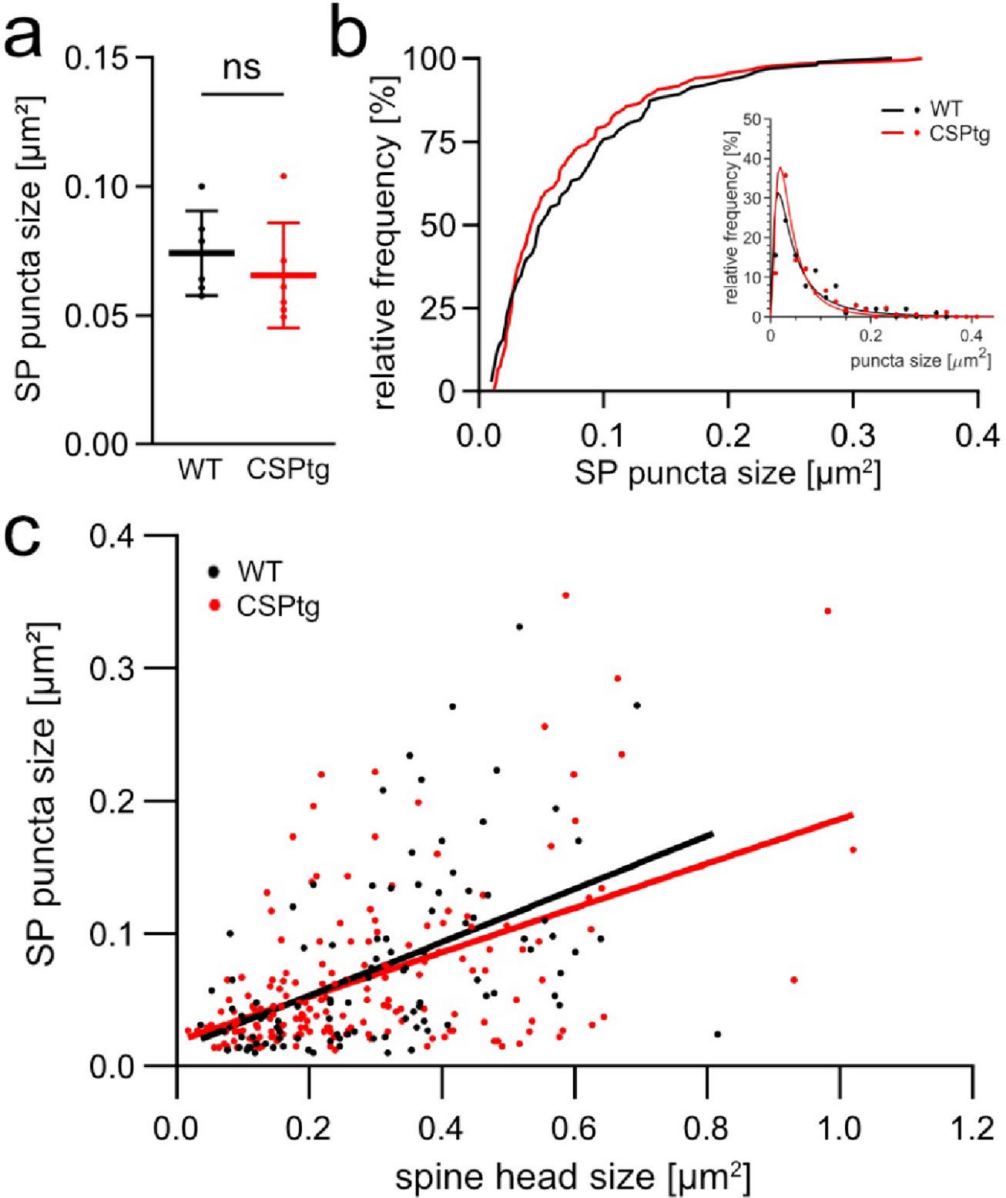
The relationship between Synaptopodin puncta size and spine head size is maintained in dentate granule cells of Cyan Fluorescent Protein-Synaptopodin transgenic mice. **(a)** Average Synaptopodin (SP) puncta size was not significantly different in wild-type (WT) and Cyan Fluorescent Protein-SP transgenic (CSPtg) granule cell spines (Mann-Whitney test: ns; *N* = 6 mice/genotype). **(b)** Distributions of SP puncta sizes for WT and CSPtg were not significantly different (Kolmogorov-Smirnov test: ns; WT: *n* = 103 puncta; CSPtg: *n* = 182 puncta; *N* = 6 mice/genotype). Inset shows the lognormal-like distributions for both datasets. **(c)** Correlation plots of SP puncta size vs. spine head size. Linear regression analysis demonstrates that the basal relationship between SP puncta size and spine head size is maintained in CSPtg (r^2^ = 0.26) compared to WT (r^2^ = 0.25).

Previous studies have shown that the size of SP puncta within spines correlates with spine head size^11,29^. Therefore, we investigated the relationship between SP puncta size and spine head size, as well as the distribution of SP puncta sizes, in both WT and CSPtg mice. On average, SP puncta sizes did not differ between genotypes (WT: 0.074 ± 0.016 vs. CSPtg: 0.066 ± 0.02 μm²; Fig. 5a). Similar results were obtained for the distributions of SP puncta sizes for WT and CSPtg (Fig. 5b). Notably, spine head size and SP puncta size were tightly correlated in CSPtg mice, as in WT mice (Fig. 5c). This indicates that the relationship between SP puncta size and spine head size is maintained in the CSPtg mouse line.

### GCs in CSPtg mice have more spines that contain SA organelles than GCs in WT mice

**Fig. 6:**
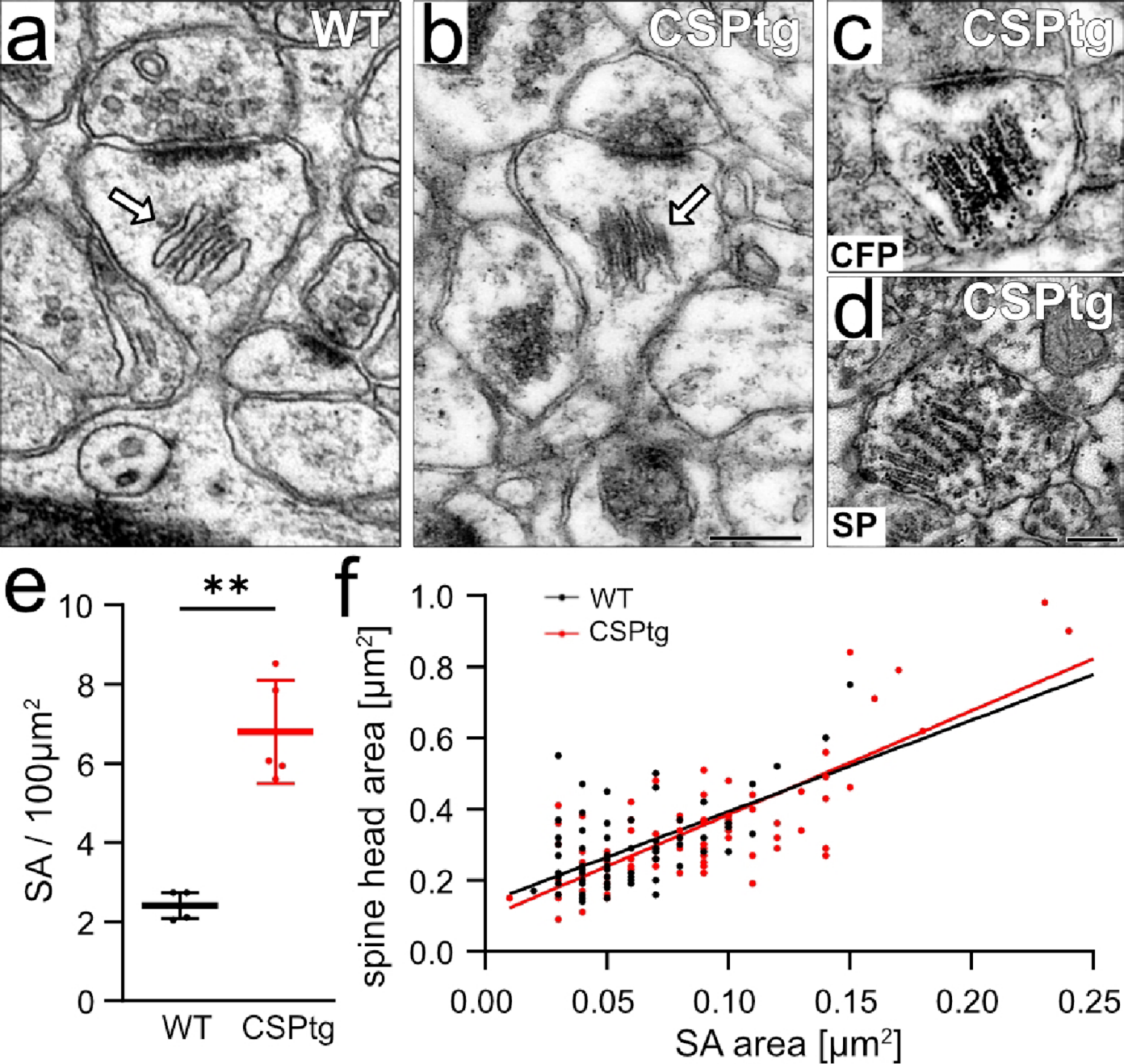
Ultrastructural analysis of granule cell spines and spine apparatuses in wild-type and Cyan Fluorescent Protein-Synaptopodin transgenic mice. **(a**,** b)** Electron microscopy of granule cell (GC) spines with a spine apparatus (SA, arrow) in the outer dentate molecular layer of wild-type (WT) and Cyan Fluorescent Protein (CFP)-Synaptopodin (SP) transgenic mice (CSPtg). **(c)** Immunolabeling for CFP shows positive SA in a GC spine of a CSPtg mouse. **(d)** Immunolabeling for SP correspondingly labels the SA in CSPtg. **(e)** The density of SAs is significantly higher in CSPtg mice compared to WT (Mann-Whitney test: *p* = 0.0079; *N* = 5 mice/genotype). **(f)** Correlation graphs of spine head area vs. SA area. Linear regression analysis demonstrates that the basal relationship between spine head area and SA area is maintained in CSPtg (*N* = 5 mice, *n* = 86 spines/SA profiles; r^2^ = 0.61) compared to WT (*N* = 5 mice, *n* = 70 spines/SA profiles; r^2^ = 0.35). Scale bars: **(a–d)** 250 nm.

Next, we investigated the SP puncta in GCs at the ultrastructural level using electron microscopy. The ultrastructure of the SA in CSPtg was identical to that of WT: cisterns and tubules of smooth ER were held together by dense plates and arranged in compact stacks (Fig. 6a, b). These formations were typically found in the head or neck of spines. Immunolabeling for CFP (Fig. 6c) and SP (Fig. 6d) verified the presence of SP_TG_ at the SA of GC spines in CSPtg.

The presence of SP puncta within spines indicates the presence of a SA organelle^25,47^. Conversely, a SA does not form in the absence of SP ^24^. We wondered whether increased levels of SP result in an increase in the number of SA organelles. To address this question, we quantified the SA density in the DG outer molecular layer (oml) and found a significant increase in SA density in CSPtg (6.8 ± 1.31 SA per 100 μm²) compared to WT (2.4 ± 0.33 SA per 100 μm²; Fig. 6e). This demonstrates that the increase in SP+ spines in CSPtg mice is accompanied by an increase in SA-containing spines. Finally, we analyzed whether the correlation between spine head size and SA size is maintained in CSPtg mice. We consider this important, as it provides further evidence of the biological function of the SP_TG_ protein. In both genotypes, we observed the same tight correlation between SA size and spine head size (Fig. 6f). Notably, we occasionally observed very large SA organelles in CSPtg mice. These SAs were larger than any previously observed in WT mice. However, there were too few of these “giants” for meaningful quantification.

### SP_TG_ rescues the SA organelle in SP-deficient mice

**Fig. 7:**
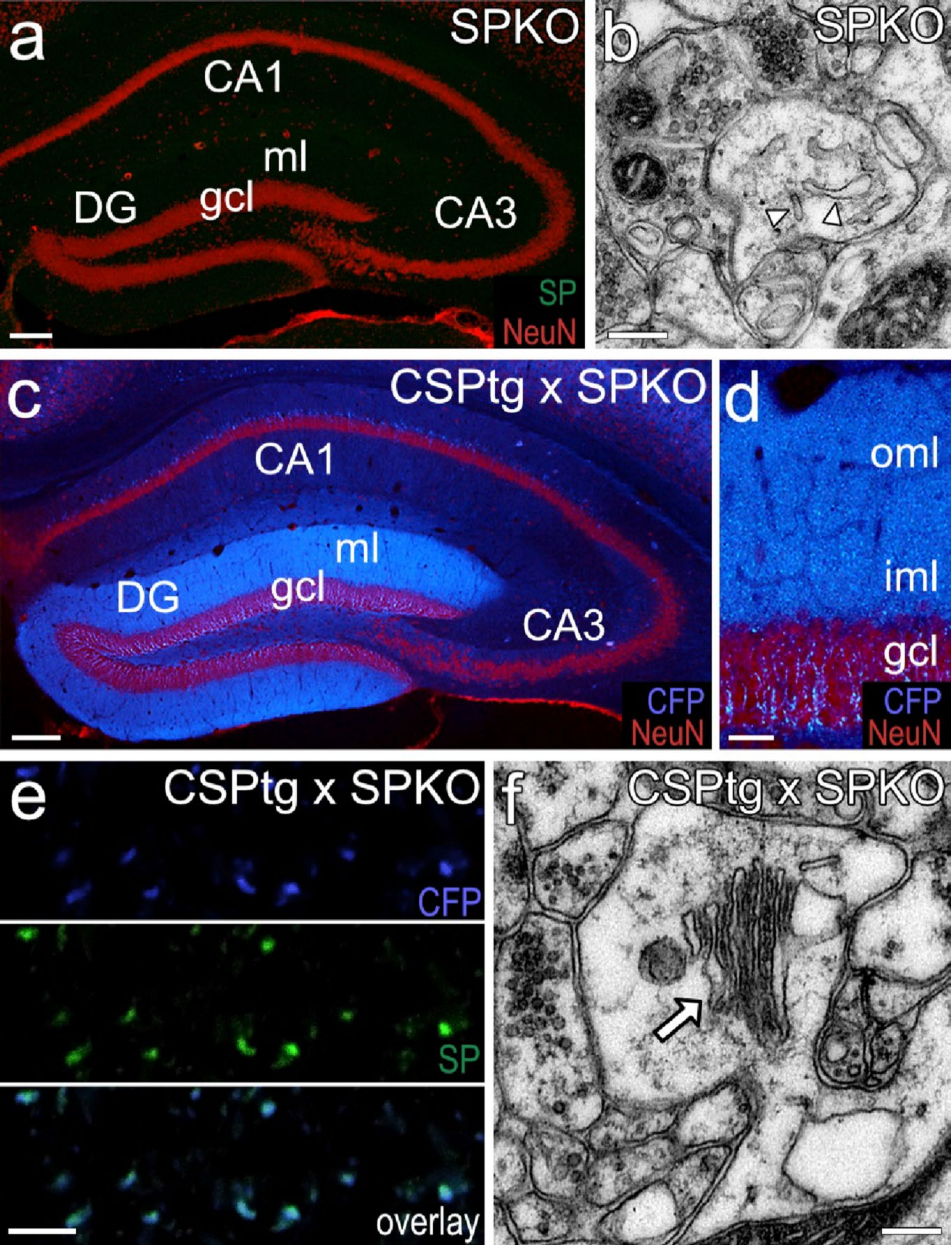
Cyan Fluorescent Protein-Synaptopodin rescues the spine apparatus in Synaptopodin-deficient granule cells. **(a)** Immunolabeling for Synaptopodin (SP) in the SP-deficient mouse (SPKO) shows no specific labeling. Section was counterstained for neuronal marker NeuN to visualize hippocampal cell layers. **(b)** Spine apparatus (SA) organelles are absent in granule cell (GC) spines of SPKO mice. Notably, large spines frequently contain tubules of unstacked ER (arrowheads). **(c)** Cyan Fluorescent Protein (CFP)-SP transgenic mice (CSPtg) were crossed with SPKO mice, generating CSPtg x SPKO mice, that express only transgenic CFP-SP. Section was counterstained for NeuN. Note the strong expression of SP in the molecular layer (ml) of the dentate gyrus (DG) compared to cornu ammonis (CA) regions CA1 and CA3. **(d)** Higher magnification of the DG. SP puncta are observed in the outer (oml) and inner molecular layer (iml), as well as in the granule cell layer (gcl). **(e)** Higher magnification of the DG ml for CFP, SP and overlay. All puncta are double-positive for CFP and SP in the CSPtg x SPKO mutant. **(f)** Electron microscopy of GC spines in the DG ml of the CSPtg x SPKO mutant revealed normally structured SA (arrow). Representative images are shown from *N* = 3 SPKO mice and *N* = 3 CSPtg × SPKO mice. Scale bars: (a, c) 100 μm, (b, f) 250 nm, (d) 25 μm, (e) 2.5 μm.

To provide further evidence that SP_TG_ is biologically functional, we crossed CSPtg mice with SP-deficient mice^24^. These CSPtg x SPKO mice lack SP_WT_ and only express SP_TG_. Compared to SPKO mice, which lack SP (Fig. 7a) and a SA organelle (Fig. 7b), CSPtg x SPKO mice showed robust SP expression in the hippocampus (Fig. 7c). In the DG, SP puncta were found in their characteristic locations: the ml and the gcl (Fig. 7d). Immunofluorescence labeling for SP revealed that all CFP-positive puncta were SP-positive, and vice versa (Fig. 7e). At the ultrastructural level, GC spines showed typical SA organelles (Fig. 7f), demonstrating that SP_TG_ can rescue the SA in the absence of SP_WT_. In summary, these findings indicate that SP_TG_ is biologically functional and can substitute for SP_WT_ with respect to SA formation and localization.

### SP overexpression increases mEPSC frequencies in GCs

**Fig. 8:**
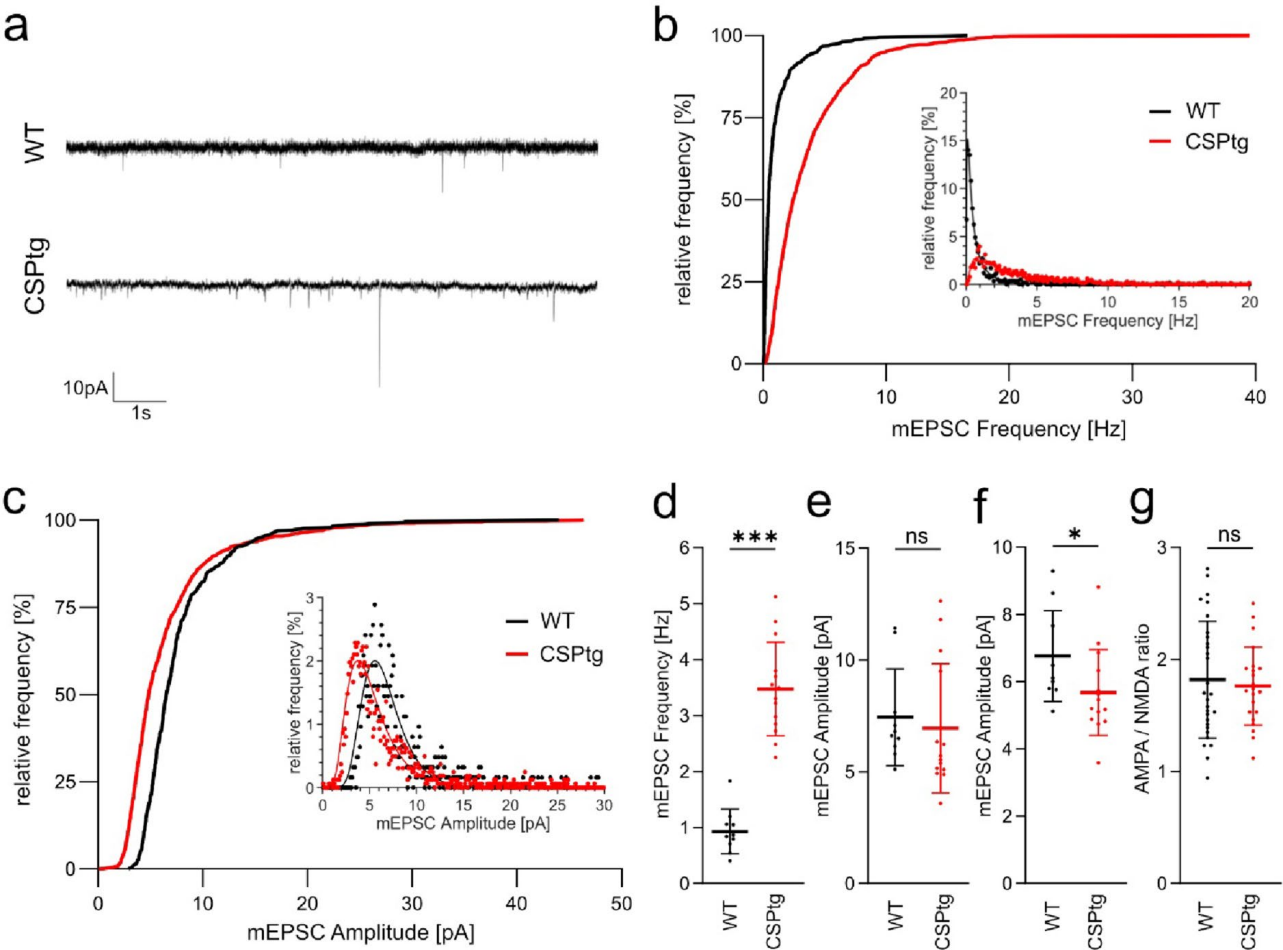
Patch-clamp recordings from granule cells of wild-type and Cyan Fluorescent Protein-Synaptopodin transgenic mice. **(a)** Examples of single traces recorded from wild-type (WT) and Cyan Fluorescent Protein-Synaptopodin transgenic (CSPtg) mice. **(b)** Cumulative distribution of miniature excitatory postsynaptic current (mEPSC) frequencies. The inset shows the fitted lognormal distribution for comparison. The cumulative frequency distribution differed significantly between genotypes, with higher mEPSC frequencies in CSPtg (Kolmogorov-Smirnov (K-S) test: *p* < 0.001; WT: *n* = 10 cells, 593 events; CSPtg: *n* = 14 cells, 1515 events). **(c)** Cumulative distribution of individual mEPSC amplitudes. The inset shows the fitted lognormal distribution for comparison. The event-level distribution differed significantly between genotypes and was shifted toward smaller amplitudes in CSPtg (K-S test: *p* < 0.001; WT: *n* = 10 cells, 623 events; CSPtg: *n* = 14 cells, 1618 events). **(d)** Mean mEPSC frequency was significantly higher in CSPtg compared to WT (Mann-Whitney (M-W) test: *p* < 0.001; WT: *n* = 10 cells from *N* = 3 animals; CSPtg: *n* = 14 cells from *N* = 3 animals). **(e)** Mean mEPSC amplitude did not differ significantly between CSPtg and WT (M-W test: ns; WT: *n* = 10 cells from *N* = 3 animals; CSPtg: *n* = 14 cells from *N* = 3 animals). **(f)** Mean amplitudes of small mEPSCs (< 15 pA) were significantly lower in CSPtg compared to WT (M-W test: *p* = 0.031; WT: *n* = 10 cells from *N* = 3 animals; CSPtg: *n* = 14 cells from *N* = 3 animals). **(g)** AMPA/NMDA ratio in CSPtg compared to WT was not significantly different (M-W test: ns; WT: *n* = 29 cells from *N* = 3 animals; CSPtg: *n* = 22 cells from *N* = 3 animals).

Next, we investigated the functional implications of the increased SP+ spine and SA density in CSPtg mice. Since SP has been associated with synaptic strength and high AMPAR densities at spine heads^10,15^, we examined mEPSC frequencies and amplitudes in GCs of WT and CSPtg mice (Fig. 8a). The cumulative distribution of mEPSC frequencies was shifted toward higher values in CSPtg mice compared with WT controls (Fig. 8b). In addition, the cumulative distribution of individual mEPSC amplitudes showed a shift toward smaller amplitudes in CSPtg mice at the event level (Fig. 8c). Mean mEPSC frequency was significantly increased approximately 3.5-fold in CSPtg (Fig. 8d; WT: 0.93 ± 0.40 Hz vs. CSPtg: 3.47 ± 0.84 Hz). In contrast, mean mEPSC amplitude did not differ between WT and CSPtg (Fig. 8e; WT: 7.43 ± 2.17 pA vs. CSPtg: 6.94 ± 2.88 pA). However, when considering only the small mEPSCs, i.e. amplitudes < 15 pA, a difference between the two genotypes was detected, revealing a significant decrease in mEPSC amplitudes in CSPtg (Fig. 8f; WT: 6.76 ± 1.35 pA vs. CSPtg: 5.68 ± 1.28 pA). No difference in the AMPA/NMDA ratio was found between genotypes (Fig. 8g; WT: 1.82 ± 0.52 vs. CSPtg: 1.76 ± 0.35). Interestingly, we occasionally observed unusually large mEPSCs, which appeared more frequently in CSPtg than in WT mice. It is tempting to speculate that exceptionally large SA-containing spines might be related to such large synaptic events (cf. Figure 8a).

In sum, our findings suggest that increased SP expression in GCs results in more of the small mEPSC events as well as occasional very large events, in line with our morphological observations.

## Discussion

GC ensembles in the DG encoding contextual memories express Arc and SP ^39,40,50^. Following placement of mice into a new context, e.g. a novel environment, a new GC ensemble forms, which upregulates Arc and SP ^39^. To understand the cellular consequences of an upregulation of SP in single GCs, we used a gain-of-function approach and studied the structure and function of mouse GCs expressing higher levels of *SP* mRNA and SP protein.

The results of our study can be summarized as follows: (1) Adult CSPtg mice overexpress SP in dentate GCs. (2) The distribution pattern of SP in the DG of CSPtg mice is similar to WT. (3) SP_TG_ is biologically functional and sorted into the spine compartment, where it contributes to the formation of a SA organelle. (4) CSPtg mice have twice as many SP+ spines as WT mice. The distribution of SP+ spines in CSPtg mice shows a trend towards spines with smaller head sizes. (5) At the ultrastructural level, the increased number of SP+ spines is mirrored by a ~threefold increase in SA density. (6) At the synaptic level, an increase in mEPSC frequency is observed, raising the possibility that an increase of spines containing a spine apparatus increases the number of active synapses. In the context of GCs upregulating SP, e.g., in a novel environment, we hypothesize that increased levels of SP could increase the number of synapses available for plasticity. In turn, this could facilitate the integration of GCs into new ensembles encoding for a specific environment.

### The CSPtg mouse is an in vivo gain-of-function model

The CSPtg mouse used in our study expresses SP_TG_ under the Thy1.2 promoter. This promoter is well-established^43^ and results in strong neuronal expression of the transgene in the adult brain. Consistent with this, *SP* mRNA and SP protein were strongly expressed in dentate GCs. The distribution pattern of SP protein was similar to what has been previously described in WT: a punctate pattern in the DG ml, indicative of SP in GC spines^25,28,48^. At the level of single, identified GCs, SP is sorted into the spine compartment. Immunolabeling for CFP at the ultrastructural level verified the tight association between SP_TG_ and the SA organelle. Finally, the correlation between spine head size and SA size was maintained in CSPtg mice. In sum, these findings suggest that SP_TG_ is expressed in a WT-like pattern in CSPtg mice and is correctly sorted to spines, where it is integrated into the SA organelle.

Since the CSPtg mouse contains SP_WT_, we verified that SP_TG_ sorting into spines does not depend on the wild-type protein. Therefore, we crossed the CSPtg mouse onto a SP-knockout background. As a result, the proper SP puncta pattern was restored in the SP-deficient mouse. Furthermore, SP_TG_ rescued SA formation, demonstrating its biological functionality. Notably, all GCs appear to express SP, as revealed by fluorescence in situ hybridization for *SP* mRNA. Similarly, all GCs appeared to express CFP-SP in CSPtg x SPKO mice. Thus, dentate GCs in the CSPtg mouse line do not show a relevant mosaic expression, which has been previously reported for some transgenic lines using the Thy-1.2 promoter^51,52^.

### Higher SP levels in GCs increase the density of SP+ spines containing SAs

The in-depth characterization of GCs in CSPtg mice made us confident that this line is an appropriate gain-of-function model to understand better the role of SP in dendritic spines in the adult brain. Our results complement the findings of earlier studies that used loss-of-function strategies^10,24,29,30,53^. Consistent with the data from SP-deficient mice showing no changes in spine density^24,30,53^ or average spine head size^29^, CSPtg mice also showed no changes in these parameters. Although SP overexpression did not affect the average size of spine heads, the fraction of SP+ spines showed a strong trend towards an altered size distribution. CSPtg mice appeared to have more small SP+ spines, i.e. spines not normally containing SP or a SA organelle.

As SP is an essential component of the SA^21,24,25^, we investigated whether an increase in the number of SP+ spines also translates into an increased number of SAs. Indeed, we found ~threefold more SAs in single ultrathin sections with a normal head size-to-SA area ratio. The SA was morphologically indistinguishable from that found in wild-type and maintained a normal head size-to-SA area ratio. The slight difference in numbers (twofold increase in SP+ spines vs. threefold increase in SA) can be attributed to the higher sensitivity of EM to detect very small SAs that might escape light-microscopic detection as SP-positive puncta. Also, the single section approach used in our study has its own limitations, e.g. resulting in a detection bias of SA. To obtain a more precise estimate of SA numbers in the CSPtg mice, EM stereology will be needed. Nevertheless, our EM data confirm that overexpression of SP in granule cells increases the number of SA organelles within the same range as observed in the light microscope.

### The role of SP in structural spine plasticity

So far, SP has been linked to local calcium stores^13,15,30,31^, AMPAR trafficking to the postsynaptic compartment of excitatory synapses^15^, synaptic strength^15^, and long-term spine stability^29^. However, it has not been fully elucidated at which stage or stages during structural spine plasticity SP plays a role and how and when SP is integrated into the spine compartment.

Structural plasticity has been subdivided into three phases: an initial phase in which the spine expands; a transient phase in which the spine head shrinks again; and a sustained phase in which the size of the spine head is stabilized^54,55^. SP has been suggested to play a role during the late phase^49,56,57^. Short-term time-lapse imaging has shown that SP enters spines only after the spine head has expanded^49^. Following stimulation, ER tubules enter spines with the help of calmodulin-associated myosin V. In caldendrin-containing spines, myosin V-caldendrin interactions form an F-actin tether that stabilizes the ER. In the final step of this process, SP enters the spines, where it joins with the ER to form the SA^56,57^. Thus, SP appears to follow spine head expansion but does not seem to be involved in its regulation. In addition to forming the SA organelle, SP has been shown to stabilize actin directly^20,21,26,27^, as well as indirectly by protecting actin filaments and Rho GTPase family members from degradation^36,49,58,59^. Taken together, these observations suggest that SP stabilizes the actin pool and, in line with this, SP+ spines were found to be considerably more stable than SP- spines of equal size^29^. The functions of SP, both as a SA organelle organizer and as an actin-stabilizing molecule, converge towards a role for SP/SA during the late phase of structural spine plasticity: The presence of SP/SA in spines makes spines more stable and their synapses stronger than spines of equal size lacking SP/SA.

### Increased availability of SP increases the number of active synapses

SP influences the strength of synapses via its role as an organizer of the SA, which acts as a subcellular calcium source and sink^13,15,30,31^. Furthermore, SP promotes AMPAR trafficking to the postsynaptic compartment of excitatory synapses^15^. Using glutamate-uncaging at SP+ and SP- spines of equal size, Vlachos et al. (2009) showed that SP+ spines are significantly stronger and that they contain more AMPARs. Therefore, increasing the fraction of SP+ spines should increase the strength of some synapses. Interestingly, we observed a trend towards small SP+ spines. Small spines have fewer AMPARs and, in some cases, the density of AMPARs on these spine heads may be so low (or absent) that they are effectively “silent”^60,61^. This raises the intriguing possibility that the insertion of SP into small “silent” spines may increase their postsynaptic AMPAR density, rendering them functionally detectable and effectively “unsilencing” them.

Our electrophysiological data from GCs in acute slices of adult CSPtg mice are compatible with this hypothesis. Compared to WT, CSPtg mice exhibited significantly higher mEPSC frequencies. Since mEPSC frequencies reflect the number of active events at synapses, this finding is consistent with an increased number of postsynaptic structures responding to spontaneous neurotransmitter release. Furthermore, we detected an increase in small synaptic events, consistent with a higher proportion of weak synapses in CSPtg mice. Of note, the AMPA/NMDA ratio was not significantly altered, indicating that this ratio is homeostatically maintained under conditions of SP overexpression. In sum, our data suggest an increased number of active synapses in CSPtg mice. However, whether the increased number of active synapses directly depends on the integration of a SA organelle into “silent spines” which are thus “unsilenced”, requires direct proof and further investigations.

### An increase in small SP+ spines may facilitate network integration of GCs

Increased levels of SP in GCs are observed under physiological, i.e., behavioral, conditions: Following placement of a mouse into a new environment, a small percentage of GCs (~ 1%) becomes activated and co-expresses Arc and high levels of *SP* mRNA^39^. These activated GCs form a neuronal ensemble that encodes for the current environmental “context”^40,41,50,62,63^. If the mouse is placed into yet another environment, a new ensemble of Arc- and SP-expressing GCs forms^39^. Although our gain-of-function approach does not directly mirror these behavioral conditions, it provides first insight into the effects an upregulation of SP has on GCs in vivo. As SP overexpression increases the number of SP+ spines and the number of active synapses of GCs, we propose that this may facilitate the integration of activated GCs into a new network. Immature and transient spines sample their environment for potential partners and appear to be part of a pool of spines available for potentiation and stabilization^60^. Increasing the availability of SP in GCs may make more synapses available for potentiation and plasticity. Furthermore, upon potentiation and spine head expansion, the presence of SP may stabilize the expanded spine head^29,49^ and may impart long-term stability to the spine^29^. Thus, the upregulation of SP in activated GCs may contribute to the integration of GCs into a new ensemble by strengthening and stabilizing their rewired synapses.

## Methods

### Ethics statement

Animal care and experimental procedures were performed in accordance with German law on the use of laboratory animals (Animal Welfare Act, TierSchG) and were approved by the Regierungspräsidium Darmstadt (permit numbers: F6/Anz. 03; V54-19c20/15-F40/30) and declared to the institutional animal welfare officer. Experiments involving animals were reported in accordance with ARRIVE guidelines.

### Animals

Male CSPtg mice bred on a C57BL/6J background, male C57BL/6J littermate control mice, male SP-deficient (SPKO) mice^24^ bred on a C57BL/6J background, and male CSPtg mice crossed onto a SPKO background (CSPtg x SPKO) were analyzed at 3–6 months of age, unless otherwise stated. Animals were bred and maintained in the institutional animal facility (Zentrale Forschungseinrichtung, ZFE) of Goethe University Frankfurt under standard housing conditions.

### Generation of CSPtg mice

Generation of CSPtg mice was performed as previously described for GFP-SP transgenic mice^10^, with modifications. In brief, a plasmid containing the open reading frame of mouse brain SP (NM_001109975) fused to the 3’ end of EGFP was generously provided by Dr. Peter Mundel. The SP open reading frame was subcloned into pECFP-C1 (Clontech) via SalI and SacII to obtain pECFP-SP. For neuron-specific expression the ECFP-SP sequence was excised using Eco47III and SmaI, and ligated into the blunted XhoI site of pThy1.2 (generously provided by Dr. Pico Caroni)^43^. The Thy1.2-ECFP-SP expression cassette was excised with NotI and PvuI, and injected into mouse (C57BL/6J) pronuclei (Gutenberg University Mainz, Germany).

### Fluorescence in situ hybridization

Mice were killed with an overdose of pentobarbital (500 mg/kg body weight) and transcardially perfused with 0.9% sodium chloride (NaCl) followed by 4% paraformaldehyde (PFA) in 0.1 M phosphate buffered saline (PBS, pH 7.4). Brains were postfixed for 4 h in 4% PFA followed by 20% sucrose at 4 °C overnight (o/n), cut into 40 μm thick sections using a cryostat (CM3050S, Leica) and stored at -20 °C until use. For fluorescence in situ hybridization, sections were incubated in 10 mM citrate buffer (pH 6) for 20 min at 85 °C, washed several times in 2 x SSC (LONZA AccuGEN) and pre-hybridized for 2 h at 60 °C with hybridization buffer (50% formamide, 5 x SSC, 5% dextran sulfate, 500 µg/ml DNA MB grade from sperm (Roche), 250 µg/ml t-RNA (Sigma Aldrich) and 1x Denhardt’s (Sigma Aldrich). After heat-treatment of a probe specific for *SP* mRNA^27^ for 5 min at 85 °C, sections were incubated with the probe in hybridization buffer (1:1000) o/n at 60 °C. Several washing steps (2 x SSC for 10 min at room temperature (RT), 2 x SSC / 50% formamide (AppliChem) for 15 min at 60 °C, 0.1 x SSC / 50% formamide for 15 min at 60 °C, 0.1 x SSC for 15 min at 60 °C and TN buffer (0.1 M Tris-HCl, 0.15 M NaCl; pH 7.4) for 5 min at RT were performed before blocking solution (1% blocking reagent (Roche) in TN buffer) was added for 30 min at RT. After blocking, anti-Digoxigenin-POD (1:2000 in blocking solution; Roche) was added to sections for 2 h at RT. Sections were washed several times with TNT (0.1 M Tris-HCl, 0.15 M NaCl, 0.3% Triton X-100) and incubated in TSA-Plus Cyanine 3 System (Perkin Elmer) diluted in amplification solution (1:50) for 10 min in the dark at RT, washed several times with TNT and mounted in Fluorescence Mounting Medium (Dako, Agilent Technologies).

### Immunofluorescence

After perfusion with 0.9% NaCl followed by 4% PFA in 0.1 M PBS (pH 7.4), brains were postfixed in 4% PFA o/n at 4 °C. Frontal sections (40 μm) of the dorsal hippocampus were cut with a vibratome (VT1000 S, Leica). Free-floating sections were washed several times in 50 mM Tris-buffered saline (TBS) containing 0.1% Triton X-100, incubated in a blocking buffer (0.5% Triton X-100, 5% bovine serum albumin (BSA) in 50 mM TBS) for 30 min at RT and subsequently incubated with the appropriate primary antibody diluted in 0.1% Triton X-100, 1% BSA in 0.05 M TBS (or diluted in 0.5% Triton X-100, 1% BSA in 0.05 M TBS for intracellular injections, see below) for 2–3 days at RT. Rabbit anti-SP (1:2000, Synaptic Systems); guinea pig anti-SP (1:2000; Synaptic Systems); mouse anti-NeuN (1:500, Chemicon) and goat anti-GFP (1:1000, Acris; which also labels CFP) were used as primary antibodies. After several washing steps, sections were incubated with the appropriate secondary Alexa-conjugated antibodies (1:2000, Invitrogen) for 4 h at RT, and finally mounted in Fluorescence Mounting Medium (Dako, Agilent Technologies) or Vectashield Antifade Mounting Medium (Linaris). Images were obtained using an EZ-C1 (Nikon) or Olympus FV1000 (Olympus) confocal microscope.

### Electron microscopy

Mice were killed with an overdose of pentobarbital (500 mg/kg body weight) and transcardially perfused with either 0.9% NaCl followed by 4% PFA and 0.5% glutaraldehyde (GA; Polysciences) in 0.1 M cacodylate buffer (pH 7.4) or 2% PFA and 6% GA for optimal ultrastructure preservation. Brains were postfixed in 4% PFA, 0.5% GA in 0.1 M cacodylate buffer at 4 °C o/n. Serial 50 μm thick frontal brain sections were cut with a vibratome (Leica VT1000S), washed in TBS, incubated in 0.1% NaBH4 (Sigma-Aldrich), and blocked with 5% BSA for 1 h at RT to reduce non-specific staining. For primary antibodies, rabbit anti-SP (1:1000, Synaptic Systems) and anti-GFP (1:1000, DPC; which also labels CFP) were used. Biotinylated specific anti-IgG (1:200; Vector Laboratories) was used as secondary antibody. After washing in TBS, sections were incubated in avidin–biotin–peroxidase complex (ABC-Elite; Vector Laboratories) for 90 min at RT and were reacted with diaminobenzidine (DAB) solution (Vector Laboratories) at RT. Some sections were silver-intensified by incubation in 3% hexamethylenetetramine (Sigma-Aldrich), 5% silver nitrate (AppliChem), and 2.5% di-sodium tetraborate (Sigma-Aldrich) for 10 min at 60 °C, in 0.05% tetrachlorogold (AppliChem) solution for 3 min, and in 2.5% sodium thiosulfate (Sigma-Aldrich) for 3 min. After staining, sections were washed in 0.1 M cacodylate buffer, osmicated (0.5% OsO4 in cacodylate buffer), dehydrated using 1% uranyl acetate (Serva) in 70% ethanol, and embedded in Durcupan (Sigma-Aldrich). Ultrathin sections were cut using a Leica Ultracut UTC and collected on single-slot Formvar-coated copper grids that were contrasted with lead citrate for 4 min.

### Spine head area and SA analysis

For this analysis, a perfusion protocol containing 2% PFA and 6% GA was used. Samples were randomly selected from comparable regions of the DG oml. The same sampling strategy and identification criteria were applied to both genotypes. The analysis was done with the analyst blinded to the genotype. Images of the oml of WT and CSPtg mice were captured with an electron microscope (Zeiss EM 900) at 20,000 x magnification. Spine head area and area of corresponding SA were manually outlined with the Polygon tool and quantified using ImageSP Viewer (SysProg). Quantification of SA densities (i.e., the number of SAs per 100 μm²) in the oml was performed at 12,000x magnification. A total of 20 images (8.56 μm x 8.56 μm each) per animal/genotype were analyzed. A SA was considered to be present within a spine if at least two stacked ER cisterns and a dense plate could be identified in a spine head. The analysis was done with the analyst blinded to the genotype.

### RNA isolation and reverse transcription-quantitative PCR

Mice were killed with an overdose of isoflurane (Abbott), brains were rapidly removed from the cranium, and the hippocampus was dissected. Tissue was immediately disrupted with a pestle homogenizer in 300 µl RLT buffer with ß-mercaptoethanol (RNeasy Plus Mini Kit, Qiagen). Total RNA was isolated following the manufacturer’s specifications. RNA concentration was checked with the Nanodrop (Thermo Fisher Scientific), and RNA integrity was assessed using the 2100 Bioanalyzer system and RNA 6000 Nano Kit (Agilent Technologies). Total RNA was reverse transcribed using High-Capacity cDNA Reverse Transcription Reagents Kit (Applied Biosystems) following the manufacturer’s recommendations. Quantitative PCR was performed using TaqMan™ Gene Expression Assays (Applied Biosystems) for *Gapdh* (Mm99999915_g1) and *Actb* (Mm00607939_s1) as reference genes for normalization, and a custom-made TaqMan™ Gene Expression Assay for *SP* (forward primer 5′-GTCTCCTCGAGCCAAGCA-3′; reverse primer 5′-CACACCTGGGCCTCGAT-3′ and probe 5′-TCTCCACCCGGAATGC-3′) using the StepOnePlus Real-Time PCR System (Applied Biosystems) and TaqMan™ Fast Universal PCR Master Mix (Applied Biosystems). Amplicon specificity was verified using DNA 1000 Chips with the Agilent 2100 Bioanalyzer system (Agilent Technologies).

### Qualitative western blot analysis

For protein extraction, 10x volume of homogenization buffer (20 mM Tris, 500 mM NaCl, 0.5% CHAPS, 5 mM EDTA), PhosSTOP™ (phosphatase inhibitor cocktail tablets, Roche) and cOmplete™ Mini (protease inhibitor cocktail tablets, Roche) was added to freshly dissected hippocampus. Homogenization was performed with a pestle (Wheaton). After centrifugation at 4 °C for 30 min (22,000 rpm, Centrifuge 5415 R, Eppendorf) protein concentration was quantified with a Qubit^®^ 2.0 Fluorometer (Life Technologies) using Qubit^®^ Protein Assay Kit (Life Technologies). Samples were denatured for 5 min at 95 °C and immediately cooled down on ice. For gel electrophoresis, protein amounts (approximately 10 µg) were loaded onto 8% SDS–polyacrylamide gels and were separated at 120 V for 15 min followed by 180 V for 45 min. Subsequently, gels were blotted to nitrocellulose membranes at 15 V for 75 min. Blots were then washed twice in TBS and incubated with Odyssey Blocking Buffer (1:1 with TBS, LI-COR Biosciences) at 4 °C o/n. Blots were washed again in TBS and incubated o/n at 4 °C with rabbit anti-SP (1:20,000; Synaptic Systems) or goat anti-GFP (1:10,000; Acris) diluted in 1:1 Odyssey Blocking Buffer with TBS and 0.1% Tween20. Blots were washed in TBS with 0.1% Tween20 and incubated with an IRDye800CW conjugated secondary antibody (LI-COR Biosciences) at RT for 45 min. For loading control, mouse anti-beta Actin antibody (1:10,000; Abcam) in combination with an IRDye680 conjugated goat anti-mouse antibody (1:10,000; LI-COR Biosciences) was used. Two-color imaging was performed using Odyssey^®^ Infrared Imaging System (LI-COR Biosciences).

### Intracellular injections

Mice were killed with an overdose of pentobarbital (500 mg/kg body weight) and transcardially perfused with 0.1 M PBS followed by perfusion with 4% PFA for 4 min and postfixed in 4% PFA o/n at 4 °C. Frontal sections of 250 μm thickness were cut with a vibratome (VT1000 S, Leica). Sections of the dorsal hippocampus were positioned in a grounded, custom-made basin filled with 0.1 M PBS and mounted on a fixed-stage Patch Clamp Tower System (Science Products). For visualization, a BX51WI microscope (Olympus) attached to a VT-1 xy Microscope Translator (Science Products) and a 10x objective (LMPlanFLN10x, NA 0.25, WD 21 mm; Olympus) were used. Sharp quartz glass filament electrodes (QF100-70-10; O.D: 1.0 mm, I.D.: 0.7 mm; Sutter Instruments) were produced with a P-2000 laser puller (Sutter Instruments). Electrode tips were filled with 0.75 mM Alexa Fluor 568-Hydrazide (Thermo Fisher Scientific) in HPLC-grade H_2_O (VWR Chemicals, HiPerSolv CHROMANORM) and backfilled with 0.1 M LiCl in HPLC-grade H_2_O. Under visual control, the electrode was lowered into the suprapyramidal GC layer while applying a negative square voltage pulse (-1 V, 1 Hz) via a silver wire in line with a 500 MOhm resistance using a motorized 3D micromanipulator (DC-3 K, Märzhäuser). GCs were filled for at least 10 min. Sections were fixed with 4% PFA in PBS at 4 °C o/n, washed and embedded in 5% agar in 0.1 M PBS, resliced to 40 μm frontal sections using a vibratome (VT1000 S, Leica) and were further processed for SP immunolabeling. Thereafter, confocal z-stacks (0.25 μm step size) of Alexa Fluor 568-Hydrazide-filled dendritic GC segments in the oml of the dentate suprapyramidal blade were obtained using a confocal microscope (EZ-C1, Nikon) and a 60x oil immersion objective (N.A. 1.3; Nikon) with a 5x digital zoom. Images were taken 10 μm to 60 μm away from the hippocampal fissure. Images of dendritic segments with at least 20 μm in length were captured and only spines attributed to a given segment were analyzed.

### Quantification of dendritic GC spines and SP puncta

Spine density, spine head size, and SP puncta size of intracellular-injected GC dendrites stained with SP antibody were analyzed using Fiji^44^ and a pen tablet (Intuos CTH-480, Wacom). The length of a segment was determined by outlining the dendritic shaft in z-projection. A standardized protocol for spine quantification was applied, as described^29,45^. In brief, only spines/protrusions crossing a line set 0.2 μm away from the dendritic shaft by at least 1 pixel were included in the analysis and spines with a distinguishable spine head were marked. Spines were manually tracked across all z-planes and the maximum cross-sectional area (areamax; > 50% outside of the dendritic shaft border) of the spine head was quantified. The areamax of SP puncta co-localizing with identified dendritic spines was quantified using a custom-built Fiji-macro for image segmentation. SP puncta were quantified using the Particle Analyzer command (Fiji). Macro parameters were kept the same for all image stacks. Only spines with a distinguishable head were correlated to their individual SP puncta size. Spines were counted as SP+ when the maximum area of a SP puncta co-localized with the protrusion or the head and/or neck of a spine. Co-localization was verified in all 3 dimensions via the Orthogonal View-tool (Fiji) and in 3D-reconstructions. The ratio of SP+ spines to all spines found in a dendritic segment was calculated. The analysis was done on three segments/animal, for a total of 18 segments/genotype, with the analyst blinded to the genotype.

### Curve fitting of spine head sizes and SP puncta sizes

The spine head size and puncta size distributions were fitted by a lognormal probability density function f that is fully described by mean (µ) and standard deviation (σ):

$$\:f\left(x\right)=\frac{1}{x}\times\:\frac{1}{\sigma\:\sqrt{2\pi\:}}\times\:exp\left(\frac{-({ln\left(x\right)-\mu\:)}^{2}}{2{\sigma\:}^{2}}\right)$$

MATLAB R2018b software was used to incorporate the above function f as a new MATLAB fitting function (*fittype*). The fit was normalized for the area with the MATLAB function *trapz* to ensure that the integral over the entire range of spine sizes equals one. This allows for a comparison of different spine distributions in cells with different ranges of spine sizes. The fitting function estimates the best fitting set of µ and σ to describe the data and statistically evaluates the found set by calculating the coefficient of determination (R-squared, r^2^) as a measure for the goodness of fit. Kolmogorov-Smirnov (K-S) tests were used to compare cumulative spine head size distributions.

### In vitro slice preparation

Animals (8–12 weeks old) were anesthetized by intraperitoneal injection of ketamine (250 mg/kg, Ketaset, Zoetis) and medetomidine-hydrochloride (2.5 mg/kg, Domitor, OrionPharma) prior to intracardial perfusion using ice-cold artificial cerebrospinal fluid (ACSF) consisting of the following (in mM): 50 sucrose, 125 NaCl, 2.5 KCl, 25 NaHCO_3_, 1.25 NaH_2_PO_4_, 2.5 glucose, 6 MgCl_2_, 0.1 CaCl_2_ and 2.96 kynurenic acid (Sigma-Aldrich), oxygenated with 95% O_2_ and 5% CO_2_. Tissue blocks containing the dorsal hippocampus were sectioned into 300 μm coronal slices using a vibrating blade microtome (VT1200S, Leica). Prior to the experiment, slices were kept at 37 °C for 30 min in oxygenated extracellular solution containing the following (in mM): 22.5 sucrose, 125 NaCl, 2.5 KCl, 25 NaHCO_3_, 1.25 NaH_2_PO_4_, 2.5 glucose, 1.2 MgCl_2_ and 1.2 CaCl_2_.

### In vitro patch-clamp recordings

After recovery, brain slices were placed in a heated recording chamber (34–36 °C) that was perfused with an oxygenated ACSF (with a flow rate of 2–4 ml min^− 1^) containing (in mM): 22.5 sucrose, 125 NaCl, 2.5 KCl, 25 NaHCO_3_, 1.25 NaH_2_PO_4_, 2.5 glucose, 1.2 MgCl_2_ and 2.4 CaCl_2_. Gabazine (SR95531, 4 µM) and tetrodotoxin (TTX, 500 nM) were added to inhibit GABAergic synaptic transmission and block AP-triggered synaptic release to enable selective recording of miniature excitatory postsynaptic currents (mEPSCs). Dentate GCs were visualized using infrared differential interference contrast video microscopy with a digital camera (VX55, Till Photonics) connected to an upright microscope (Axioskop 2, FSplus, Zeiss; 40x water immersion lens). Patch pipettes were pulled from borosilicate glass (GC150TF-10; Harvard Apparatus, Holliston) using a temperature-controlled, horizontal pipette puller (DMZ-Universal Puller, Zeitz). Patch pipettes (4–6 MΩ) were filled with a solution containing the following (in mM): 120 CsCH_3_SO_3_, 8 CsCl, 10 HEPES, 0.4 EGTA, 0.2 Li-GTP, 2 Mg-ATP, 1 MgCl_2_ (pH = 7.2 adjusted with CsOH, 280 mOsm adjusted with sucrose). Series resistance was < 20 MΩ and not compensated. Recordings (> 15 min) were performed using an EPC-10 patch-clamp amplifier (Heka Electronics) with a sampling rate of 20 kHz and a low-pass filter (Bessel, 5 kHz). For analysis, mEPSCs recordings were further digitally filtered at 500 Hz. We used the IGOR (Wavemetrics, Portland, US)-based NeuroMatic v3.0 toolkit^46^. To detect individual mEPSCs, we used the following settings in the NeuroMatic-event detection module: negative events, amplitude > 4 standard deviations of local baseline, search parameters: x-axis baseline window 100 ms, search window 50 ms, points to advance 50 data points. For AMPA/NMDA ratio measurements, EPSC was recorded in GCs using whole-cell patch-clamp techniques while perfusing a slice with artificial CSF. EPSC was evoked by stimulation (200 µs duration; 1–10 V intensity) of axons passing through the inner molecular layer of the DG using a concentric bipolar electrode with 20 s interstimulus interval. AMPAR- and NMDAR-mediated EPSCs were recorded at holding potentials of -70 mV and + 40 mV, respectively. The amplitudes of AMPAR-EPSCs were measured at their peak at -70 mV; the amplitude of NMDAR-EPSCs was measured at + 40 mV, 50 ms after stimulation to exclude an AMPAR-EPSC contribution. The AMPA/NMDA ratio was calculated by dividing the peak amplitude of AMPAR-EPSCs by the amplitude of NMDAR-EPSCs measured at 50 ms. The analysis was done with the analyst blinded to the genotype.

### Statistical analysis

Statistical tests, N (number of animals or cells) and n (number of observations) values are specified in figure legends. Statistical tests were chosen based on the experimental conditions and performed using Prism (GraphPad Software), MATLAB software or ‘R’ (*r-project.org*). Statistical values are expressed as mean ± standard deviation, unless otherwise stated. Significance thresholds were: ns (not significant), *p* ≥ 0.05; * *p* < 0.05; ** *p* < 0.01; *** *p* < 0.001.

## Supplementary Information

Below is the link to the electronic supplementary material.

Supplementary Material 1

## Data Availability

The data that support the findings of this study are available from the corresponding author upon reasonable request. Electrophysiological data are available from JR upon reasonable request.
