# Supplementary material for: Overexpression of Synaptopodin increases the number of spine apparatuses and active synapses of dentate granule cells
Source: Sci Rep. 2026 Jun 11;16:18175. doi: 10.1038/s41598-026-57346-z (PMC13260461; doi:10.1038/s41598-026-57346-z)
Supplement: Supplementary file 1 — Supplementary Material 1 [file 41598_2026_57346_MOESM1_ESM.pdf]

Supplementary Figure S1.

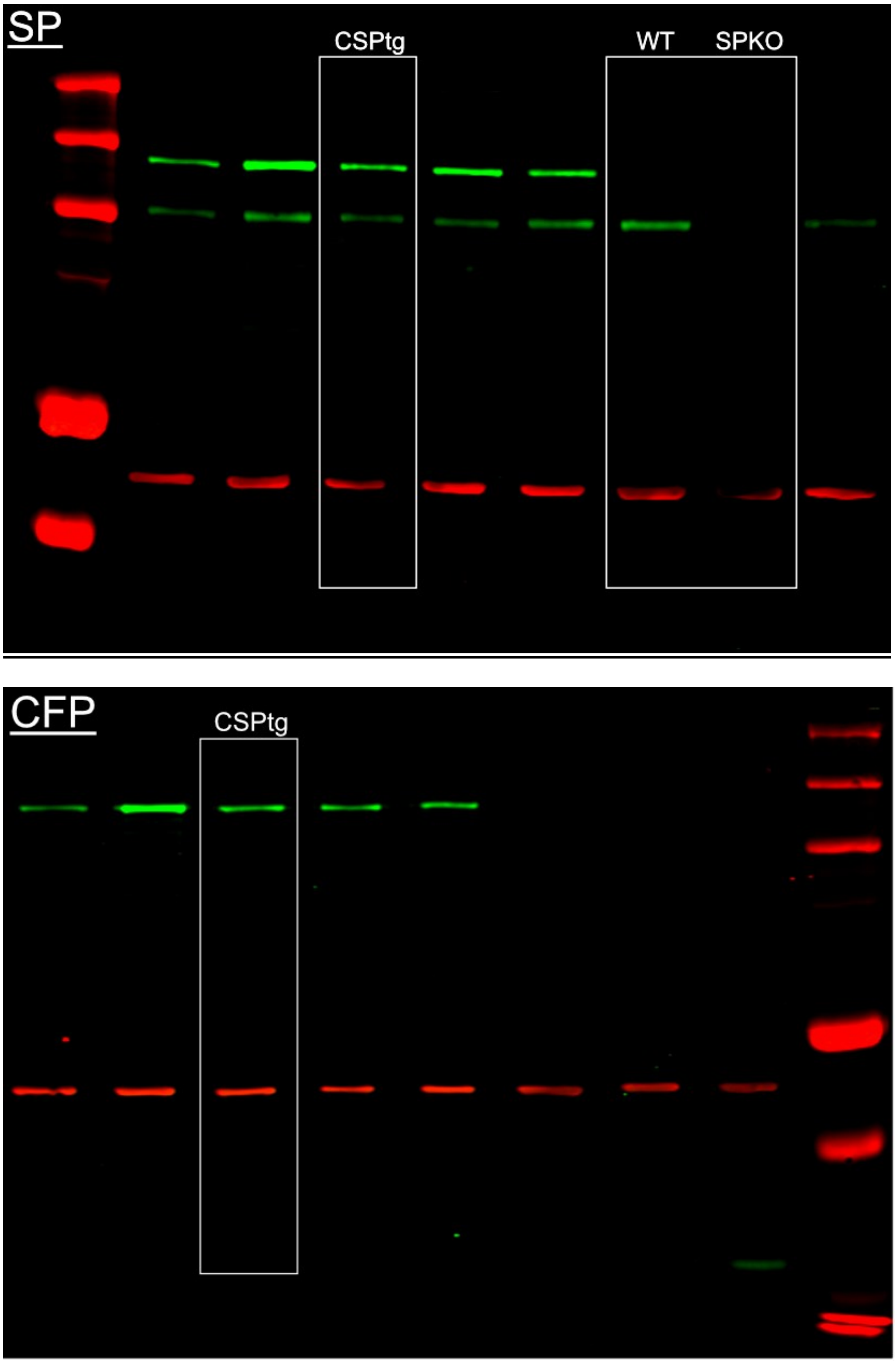

Full-length original blots corresponding to Figure 1b.

Boxed lanes correspond to the samples shown in Figure 1b. Additional lanes contain samples or controls not included in the main figure and are shown for transparency.
